# The IL-2 SYNTHORIN molecule promotes functionally adapted Tregs in a preclinical model of type 1 diabetes

**DOI:** 10.1172/jci.insight.182064

**Published:** 2024-12-20

**Authors:** Fernando Alvarez, Nicole V. Acuff, Glenn M. La Muraglia, Nazila Sabri, Marcos E. Milla, Jill M. Mooney, Matthew F. Mackey, Mark Peakman, Ciriaco A. Piccirillo

**Affiliations:** 1 Department of Microbiology and Immunology, McGill University, Montreal, Quebec, Canada.; 2 Program in Infectious Diseases and Immunology in Global Health, the Research Institute of the McGill University Health Centre (RI-MUHC), Montreal, Quebec, Canada.; 3 Centre of Excellence in Translational Immunology (CETI), RI-MUHC, Montreal, Quebec, Canada.; 4 Synthorx, a Sanofi company, La Jolla, California, USA.; 5 Sanofi, Cambridge, Massachusetts, USA.

**Keywords:** Autoimmunity, Therapeutics, Autoimmune diseases, Cytokines, T cells

## Abstract

Deficits in IL-2 signaling can precipitate autoimmunity by altering the function and survival of FoxP3^+^ regulatory T cells (Tregs) while high concentrations of IL-2 fuel inflammatory responses. Recently, we showed that the non-beta IL-2 SYNTHORIN molecule SAR444336 (SAR’336) can bypass the induction of autoimmune and inflammatory responses by increasing its reliance on IL-2 receptor α chain subunit (CD25) to provide a bona fide IL-2 signal selectively to Tregs, making it an attractive approach for the control of autoimmunity. In this report, we further demonstrate that SAR’336 can support non-beta IL-2 signaling in murine Tregs and limit NK and CD8^+^ T cells’ proliferation and function. Using a murine model of spontaneous type 1 diabetes, we showed that the administration of SAR’336 slows the development of disease in mice by decreasing the degree of insulitis through the expansion of antigen-specific Tregs over Th1 cells in pancreatic islets. Specifically, SAR’336 promoted the differentiation of IL-33–responsive (ST2^+^), IL-10–producing GATA3^+^ Tregs over other Treg subsets in the pancreas, demonstrating the ability of this molecule to further orchestrate Treg adaptation. These results offer insight into the capacity of SAR’336 to generate highly specialized, tissue-localized Tregs that promote restoration of homeostasis during ongoing autoimmune disease.

## Abstract 2

In Alvarez, we describe the immunoprotective role of the CD25-biased IL-2 SYNTHORIN™ molecule SAR444336 which promotes the generation of functionally-adapted FoxP3^+^ T_REG _in a pre-clinical model of type 1 diabetes.

## Graphical abstract

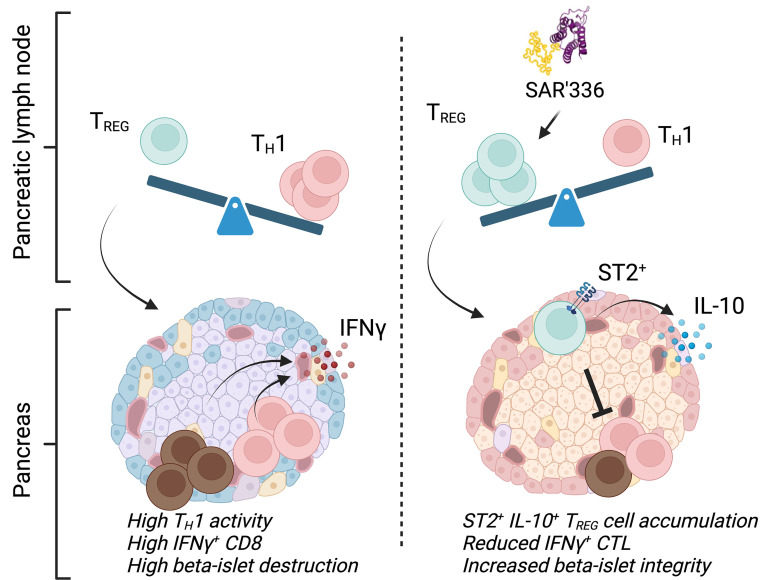

## Introduction

CD4^+^ regulatory T cells (Tregs) are a subset of T cells that possess the ability to suppress autoreactive CD4^+^ and CD8^+^ T cells and facilitate tissue repair. As such, these cells are central mediators of peripheral tolerance and are an attractive target for therapeutic intervention in autoimmune diseases. The transcriptional signature that allows Tregs to exert their suppressive functions is primarily governed by the major transcription factor FoxP3 (1, 2), the expression and function of which are induced and maintained by IL-2 through the phosphorylation of STAT5 (3). As such, IL-2 is indispensable for Treg development (3), survival (4), and function (5–7). However, one of the genes repressed by FoxP3 is the nuclear factor of activated T cells 2 (NFAT2), a promoting factor essential for the transcription of IL-2 (8), making Tregs dependent on exogenous IL-2, for which they must compete with conventional helper and effector T and NK cells. To contest for IL-2 with other immune cells, Tregs express high levels of the IL-2Rα subunit (CD25) (9), increasing the affinity of the IL-2 receptor complex consisting of IL-2Rα, β, and γ subunits for IL-2 binding (10, 11).

Many autoimmune diseases develop following a dysfunction in the ability of Tregs to access IL-2 (12). Among the better known examples of this phenomenon is type 1 diabetes (T1D), in which a decrease in the sensitivity of Tregs for IL-2 precipitates the evasion of β cell–specific, diabetogenic T cells from Treg-mediated suppression (13, 14). Studies in the nonobese diabetic (NOD) model of spontaneous T1D demonstrated that the administration of low-dose IL-2 rescues islet Tregs by supporting the expression of the antiapoptotic B-cell leukemia/lymphoma 2 protein, promoting their local proliferation, and enhancing their suppressive capacity (14, 15). Thus, stimulating IL-2 signaling in Tregs has been proposed as a viable avenue for the prevention or treatment of the disease (16–18). However, while clinical studies (19, 20) have shown that recombinant human IL-2 (aldesleukin) preferentially expands Tregs (19), off-target activation on inflammatory conventional T and NK cells was also observed, likely reflecting widespread expression of the IL-2 receptor βγ signaling complex (21). Consequently, more precise delivery of the IL-2 signal to Tregs is paramount to ensure both the safety and efficacy of this treatment in a variety of autoimmune and chronic inflammatory conditions (22).

Engineered IL-2–mutant proteins (muteins) with decreased affinity for IL-2Rβ (CD122) have been proposed as a solution to selectively target Tregs (2, 23, 24). By increasing their dependence on CD25 receptor binding, these muteins deliver a preferential phosphorylated STAT5 (p-STAT5) signal to Tregs over effector T (Teff) and NK cells (25). Nonetheless, muteins developed so far lack adequate specificity in vivo (24–26). To address this limitation, a library of site-specific covalently modified human IL-2 single variants was generated, including SAR444336 (SAR’336) (27), specifically modified by pegylation at residue H16. Modification at H16 with a 50 kDa polyethylene glycol proved particularly critical for interfering with IL-2 engagement of IL-2Rβ, while leaving binding to IL-2Rα (CD25) relatively intact, thereby retaining capacity to promote high levels of STAT5 phosphorylation (9) in CD25^hi^ T cells and making SAR’336 a compelling candidate for specifically targeting Tregs in vivo. Indeed, initial profiling demonstrated that SAR’336 potently bound CD25 without showing detectable affinity for CD122, making it capable of promoting Treg expansion and suppressive function without substantial activation and proliferation of conventional T and NK cells (27).

Little is known about the way CD25-biased IL-2 agents, like SAR’336, influence the function and nature of Tregs during autoimmunity. Similarly to conventional T cells, FoxP3^+^ Tregs adapt their transcriptional program to effectively suppress ongoing inflammation in specific tissues (28). There are numerous accounts of tissue-localized Tregs adopting master transcription factors, such as T-bet, GATA binding protein 3 (GATA3), or the RAR-related orphan receptor γ (RORγT), that provide them the necessary transcriptional signature to exert their suppressive function in tissues (29–31). We know, for example, that migrating Tregs adopt a program conferred by the major transcription factor T-bet, enabling them to express the chemokine CXCR3 to access the pancreas (32). Moreover, pancreatic Tregs express GATA3 to suppress β cell destruction in NOD mice (33). It is currently unknown whether IL-2 molecules, such as SAR’336, albeit capable of promoting Treg expansion in the periphery, influence the differentiation and functional competency of tissue-resident Tregs. Since this functional adaptation is an intricate part of pancreatic Treg function in inflammatory sites, we hypothesized that a therapeutic concentration of SAR’336 would promote differentiation of tissue-adapted Tregs during T1D disease progression in the NOD mouse model.

In this report, we aimed at defining the specificity and functional impact of SAR’336 on antigen-specific, tissue-localized Tregs, by characterizing its pancreatic and systemic effects in the context of an acute diabetogenic response in mice. We demonstrate that the CD25-biased SAR’336 molecule preferentially promotes IL-2 signaling in CD25^hi^FoxP3^+^ Tregs, not in CD8^+^ T and NK cells. In doing so, SAR’336 promotes key aspects of Treg proliferation and differentiation both in vitro and in vivo, an observation attributable to its possessing a longer half-life in medium compared with unmodified IL-2 (27). Using a NOD mouse model of T1D (34), we investigated the disease-protective effects of SAR’336 in modulating the Treg/Teff balance to control autoimmune response in islets. A single dose of SAR’336 in NOD mice increases the frequency of IL-10–producing, antigen-specific Tregs over NK cells systemically and in the pancreas and is sufficient to protect mice from disease. Moreover, SAR’336 preferentially promotes the expression of the IL-33 receptor ST2 over other tissue-associated phenotypes in Tregs, revealing a pathway by which SAR’336 drives the generation of functionally adapted, suppressive pancreatic ST2^+^ Tregs (33). Collectively, these results offer insights into the effect of an engineered, CD25^hi^ biased IL-2 molecule on the tissue adaptation of Tregs and support the therapeutic use of SAR’336 for the control of autoimmune diseases.

## Results

### A pegylated IL-2 mutein biased for CD25^hi^ T cell engagement specifically promotes FoxP3^+^ Treg expansion in vitro.

**Figure 1:**
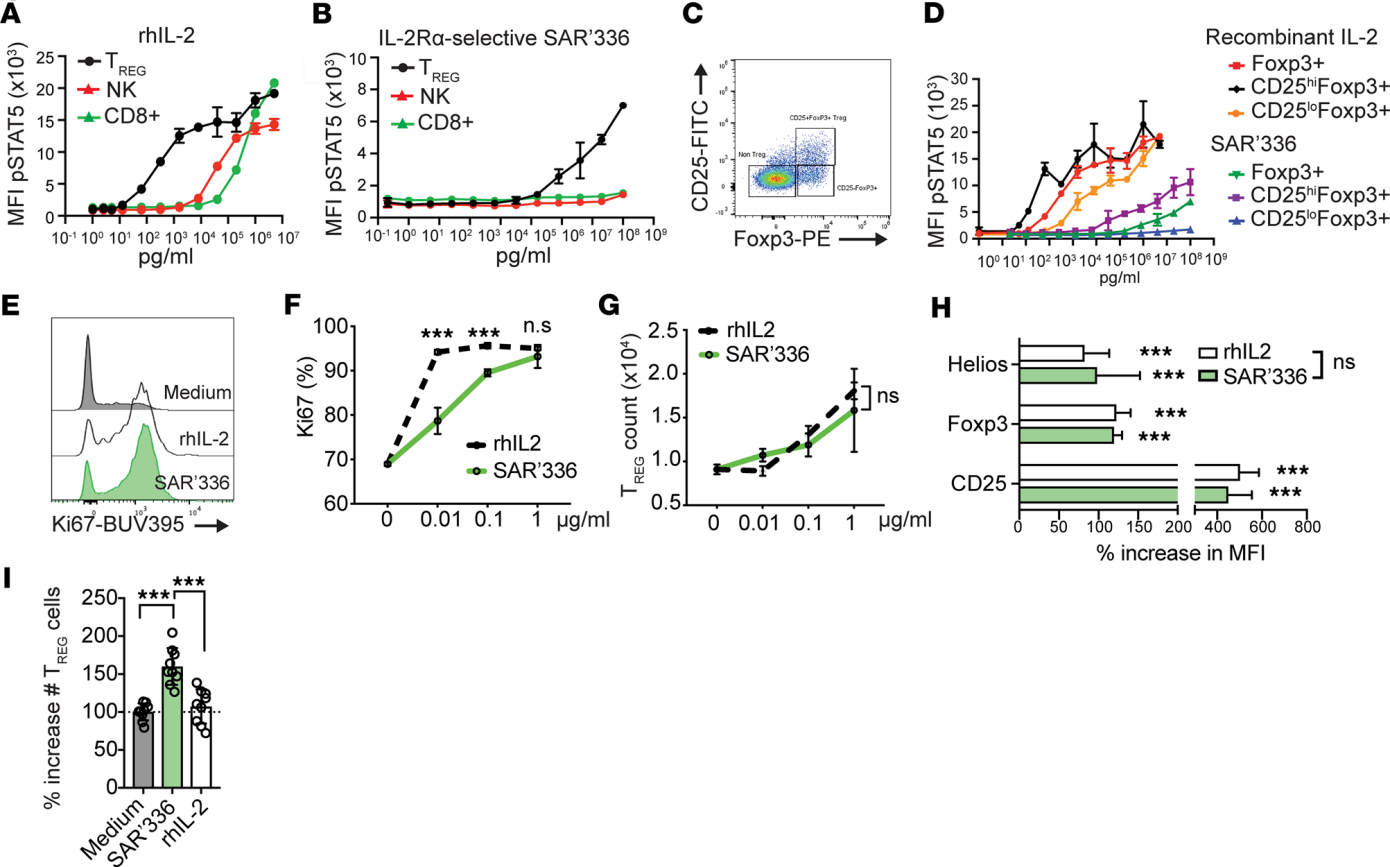
A pegylated IL-2 mutein targeting the CD25/STAT5 signaling pathway promotes specific Tregs’ expansion and Treg-associated gene expression. (**A**–**C**) Geometric mean fluorescence intensity (MFI) of phosphorylated STAT5 (pSTAT5) in FoxP3^+^CD4^+^CD3^+^ T cells (Tregs), NK1.1^+^CD3^–^ cells (NK cells), and CD8^+^CD3^+^ T cells (CD8^+^ T cells) isolated from C57BL/6 mice and exposed to increasing concentrations of rhIL-2 (**A**) or SAR’336 (**B**) for 45 minutes. Data representative of more than 1 study. (**C**) Representative flow cytometry of the gating strategy for the identification of murine CD25^hi^ and CD25^lo^ FoxP3^+^ Tregs at 45 minutes. (**D**) Effect of the concentration (pg/mL) of rhIL-2 or SAR’336 on the geometric MFI of p-STAT5 expression in total FoxP3^+^, CD25^hi^FoxP3^+^, and CD25^lo^FoxP3^+^ Tregs. The red line represents the average of FoxP3^+^ T cells in the presence of rhIL-2, and the green line represents the average of FoxP3^+^ T cells in the presence of SAR’336. (**E**–**H**) Murine CD4^+^GFP^+^ Tregs from the spleens of B6.FoxP3^GFPki^ mice were purified and activated by plated α-CD3 and α-CD28 for 72 hours in the presence of rhIL-2 or SAR’336. (**E**) Representative histogram of the expression of Ki-67 at 72 hours. (**F**) Effect of increasing dose of SAR’336 and rhIL-2 on the frequency of Ki-67^+^ among live CD4^+^FoxP3^+^ cells at 72 hours. (**G**) Effect of increasing doses in the total number of live Tregs at 72 hours. Two-way ANOVA. (**H**) Percentage increase in geometric MFI of Helios, FoxP3, and CD25 in the presence of 1 μg/mL of each cytokine/mutein over the medium alone (0 μg/mL). (Mean MFI of the experiment/mean MFI of medium alone) (*n* = 3 per experiment, 3 individual experiments). Two-way ANOVA. (**I**) CD4^+^GFP^+^ Tregs were cocultured with CellTrace Violet–labeled (CTV-labeled) CD4^+^GFP^–^ Teffs in the presence of MitoC-treated antigen-presenting cells (APCs) (CD4^–^ fraction) and soluble α-CD3 (1 μg/mL) with 1 μg/mL of rhIL-2 or SAR’336 for 72 hours. Percentage increase in the number of Tregs relative to medium: (# cells in rhIL-2 or SAR’336/mean # in medium) × 100. Compiled results of 3 distinct experiments with triplicates. One-way ANOVA. Tukey’s correction. ***P < 0.01.

The majority of FoxP3^+^ Tregs constitutively express CD25 (IL-2Rα) at relatively high levels, making them particularly sensitive to low concentrations of IL-2. However, CD8^+^ T cells and NK cells readily respond to IL-2 by expressing high amounts of the lower affinity CD122 (IL-2Rβ) chain and, as such, represent a major hurdle in the development of IL-2–modulating therapies aimed at promoting Treg-mediated immune tolerance (35). To investigate the target cell selectivity and potency of SAR’336, we exposed (CD3^+^CD4^+^FoxP3^+^) Tregs, (CD3^+^CD8^+^) CD8^+^ T cells, and (NK1.1^+^CD3^–^) NK cells isolated from the spleens of C57BL/6 mice to the mutein. Recombinant human IL-2 (rhIL-2) potently stimulated the phosphorylation of intracellular STAT5 in murine FoxP3^+^ Tregs and at higher concentrations in CD8^+^ T cells and NK cells (Figure 1A), verifying the higher sensitivity of Tregs to IL-2. On the other hand, SAR’336 specifically induced the phosphorylation of STAT5 in FoxP3^+^ Tregs, not NK or CD8^+^ T cells (Figure 1B). To verify that the signal SAR’336 provided depended on CD25 expression at the cell surface, we repeated the assay and segregated FoxP3^+^ Tregs based on the level of CD25 expression at the time of p-STAT5 staining (Figure 1C). SAR’336 preferentially induced the phosphorylation of STAT5 in CD25^hi^ Tregs, consistent with the selective nature of the mutein (Figure 1D). Thus, SAR’336, as designed (27), specifically targets CD25^hi^ Tregs, while rhIL-2 activates both CD25^–^ and CD25^+^ Tregs.

Next, we addressed the capacity of SAR’336 to promote Treg fitness in vitro. While rhIL-2 caused an increase in the expression of the mitotic marker Ki-67 in Tregs (Figure 1, E and F), SAR’336 required higher doses for all Tregs present in the culture to engage in mitosis. However, both rhIL-2 and SAR’336 led to a dose-dependent increase in the number of FoxP3^+^ Tregs by 72 hours in culture (Figure 1G). This is consistent with the fact SAR’336 requires higher concentrations to fully engage STAT5 signaling. Nonetheless, SAR’336 induced similar levels of the transcription factor Helios, FoxP3, and CD25 expression (increase in MFI) as rhIL-2 in TCR-activated cells in vitro (Figure 1H), verifying that the molecule acted as a bona fide IL-2 signal in Tregs. Helios, a transcription factor of the Ikaros family, promotes the transcriptional stability of FoxP3 in Tregs (36, 37), notably, by supporting the IL-2/STAT5 signaling pathway (38), and these results suggest Helios^+^ cells are particularly sensitive to SAR’336. Finally, since conventional effector CD4^+^ T cells (Teffs) can upregulate the CD25 receptor upon activation (39), we cocultured activated FoxP3^+^ Tregs with Teffs (1:4 ratio) to mimic a competitive environment. SAR’336, but not rhIL-2, generated increased frequencies of Tregs over Teffs (Figure 1I), demonstrating that the engineered molecule maintained its selectivity for Tregs. Collectively, these results illustrate key differences in how the pegylated SAR’336 mutein provides a p-STAT5 signal to promote the proliferation and fitness of Helios^+^CD25^hi^ Tregs.

### SAR’336 stimulates the rapid and specific expansion of FoxP3^+^ Tregs in pancreatic islets.

**Figure 2:**
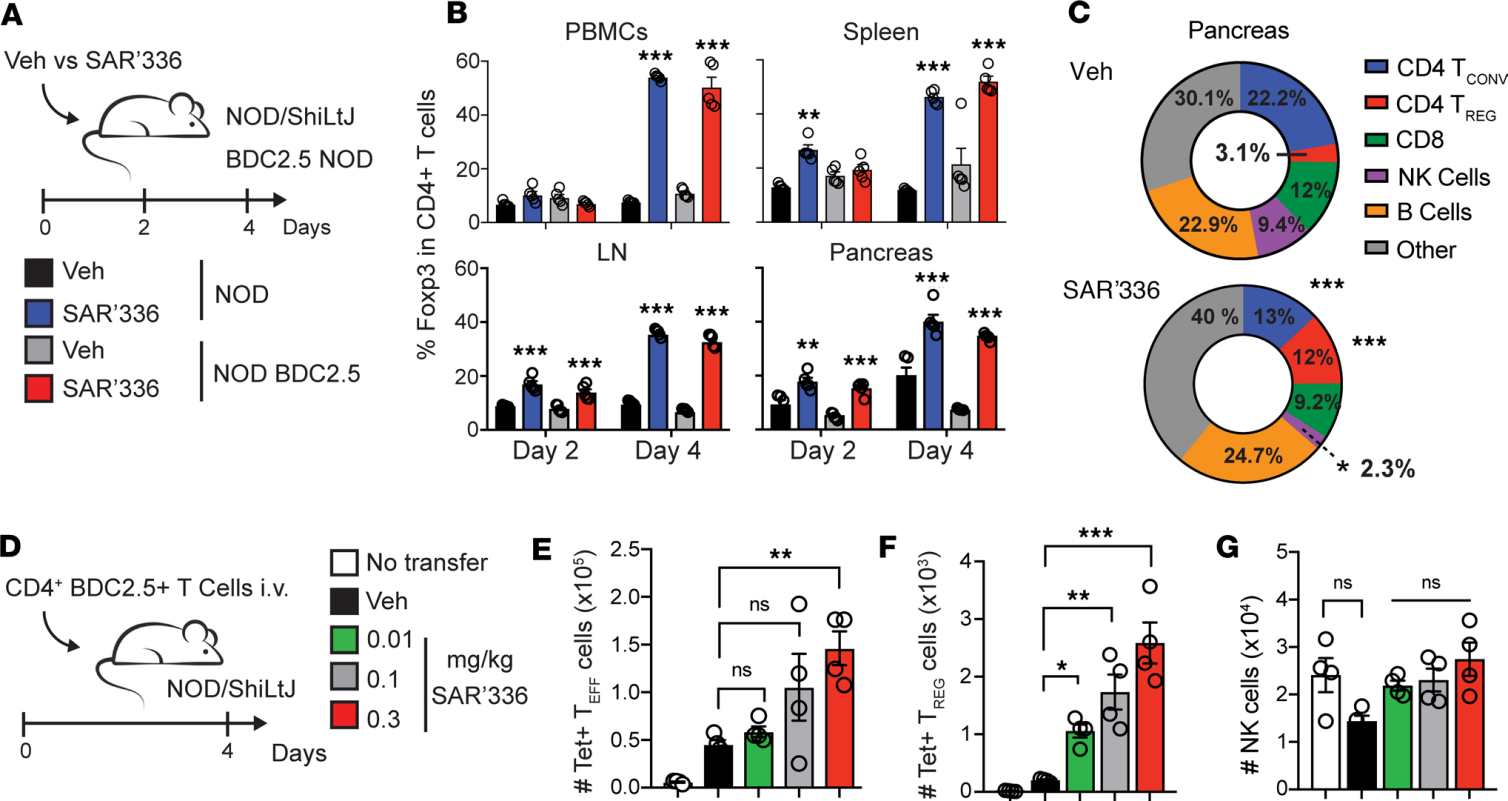
SAR’336 promotes the rapid and specific expansion of CD4^+^FoxP3^+^ T cells. (**A**) Female NOD and NOD BDC2.5 mice were administered 0.3 mg/kg of SAR’336 or the vehicle (Veh) s.c., and cells from the blood (PBMCs), the spleen, inguinal and axillary LN (pLN), and the pancreas were collected at day 2 and 4 after injection. (*n* = 4–5/group.) (**B**) Frequency of FoxP3^+^ among CD4^+^ T cells isolated in each organ at days 2 and 4 after injection. (**C**) Pie charts representing the mean frequency of conventional CD4^+^ T cells (Tconv), Tregs, CD8^+^ T cells, and NK and B cells as parts of whole cells collected from the pancreas of NOD mice at day 4 postinjection. Two-way ANOVA. *P < 0.05; **P < 0.01; ***P < 0.001. (**D** and **E**) BDC2.5^+^CD4^+^ T cells were isolated, and 5 × 10^6^ cells were adoptively transferred (i.v.) into NOD mice before the administration of 0.01, 0.1, and 0.3 mg/kg of SAR’336 or Veh s.c. (*n* = 4–5/group). (**E**) Number of BDC2.5 tetramer (Tet^+^) CD4^+^FoxP3^–^ T cells in the spleen. (**F**) Number of CD4^+^FoxP3^+^Tet^+^ cells in the spleen. (**G**) Number of NKp46^+^CD3^–^ NK cells in the spleen at day 4 after injection. One-way ANOVA. Tukey’s correction. *P < 0.05; **P < 0.01; ***P < 0.001.

It is well established that CD4^+^CD25^+^ Tregs offer protection against diabetes in the NOD model (40–42), where they depend on IL-2 to exert their suppressive functions (43). To dissect the systemic and local effects of the SAR’336 mutein on local immune responses, we administered a single dose of SAR’336 to young, female NOD and NOD BDC2.5 (nondiabetic) mice (in which CD4^+^ T cells are specific for the chromogranin A autoantigen expressed by β cells) and assessed the frequency of FoxP3^+^CD4^+^ T cells in the blood (PBMCs), spleen, peripheral lymph nodes (pLNs), and pancreas at days 2 and 4 (Figure 2A). In both groups, we observed that the frequency of Tregs among CD4^+^ T cells was highest by day 4 of injection in blood as well as in the spleen, LNs, and pancreas (Figure 2B), consistent with the recently reported pharmacodynamics of SAR’336 in wild-type C57BL/6 mice (27). Importantly, SAR’336 increased the proportion of FoxP3^+^ Tregs over Teffs and NK cells while not affecting other immune cells in the pancreas (Figure 2C). Nonetheless, in contrast with events in the pancreatic tissue, the frequency of NK cells increased slightly among circulating PBMCs (Supplemental Figure 1; supplemental material available online with this article; https://doi.org/10.1172/jci.insight.182064DS1), prompting us to investigate the off-target effects of SAR’336 in the spleen and pLNs (axillary and inguinal). Here, the number of NK cells was not increased (Supplemental Figure 2, A–E) while the ratio of Tregs to NK cells or conventional T cells was higher in the spleen (Supplemental Figure 2, B–D), verifying the preferential effect of SAR’336 on the expansion of Tregs. Finally, when we stained for the mitotic marker Ki-67, we observed that, while the frequency of Ki-67^+^ Tregs and NK cells both increased in the spleen (Supplemental Figure 2F), there were more proliferating Tregs, rather than NK cells, in the pancreas (Supplemental Figure 2G), verifying the targeted effect of the mutein on tissue-localized Tregs.

Since SAR’336 efficiently promoted the expansion of Tregs in vitro, we next investigated its effect on antigen-specific T cells in NOD mice in an acute inflammatory condition in which recently activated T cells compete for IL-2 in vivo. In this context, TCR engagement also induces CD25 expression in Teffs (39), allowing them to optimize IL-2 signals during expansion, and ultimately competing with Tregs. To address the specificity of SAR’336 in an inflammatory context in vivo, BDC2.5-specific CD4^+^ T cells were sorted and adoptively transferred into NOD mice (44), which then received vehicle or 0.3, 0.1, or 0.01 mg/kg of SAR’336 (Figure 2D). SAR’336 expanded both antigen-specific (tetramer^+^; Tet^+^) Teffs and Tregs in the spleen at the highest dose (0.3 mg/kg; Figure 2E), yet Tregs expanded at the low and intermediate dose levels of 0.01 and 0.1 mg/kg (Figure 2F), verifying that activated, antigen-specific Tregs remain more sensitive to SAR’336. Importantly, the number of recipient NK cells remained unchanged at all concentrations (Figure 2G), verifying that SAR’336 does not promote the expansion of resting NK cells. Collectively, these results highlight the selectivity of SAR’336 to target CD25^hi^ T cells and preferentially promote Treg expansion, even in an inflammatory setting where effector T cells upregulate CD25 surface expression.

### SAR’336 minimizes cytotoxic responses and expands IL-10–producing, antigen-specific Tregs in the pancreas.

**Figure 3:**
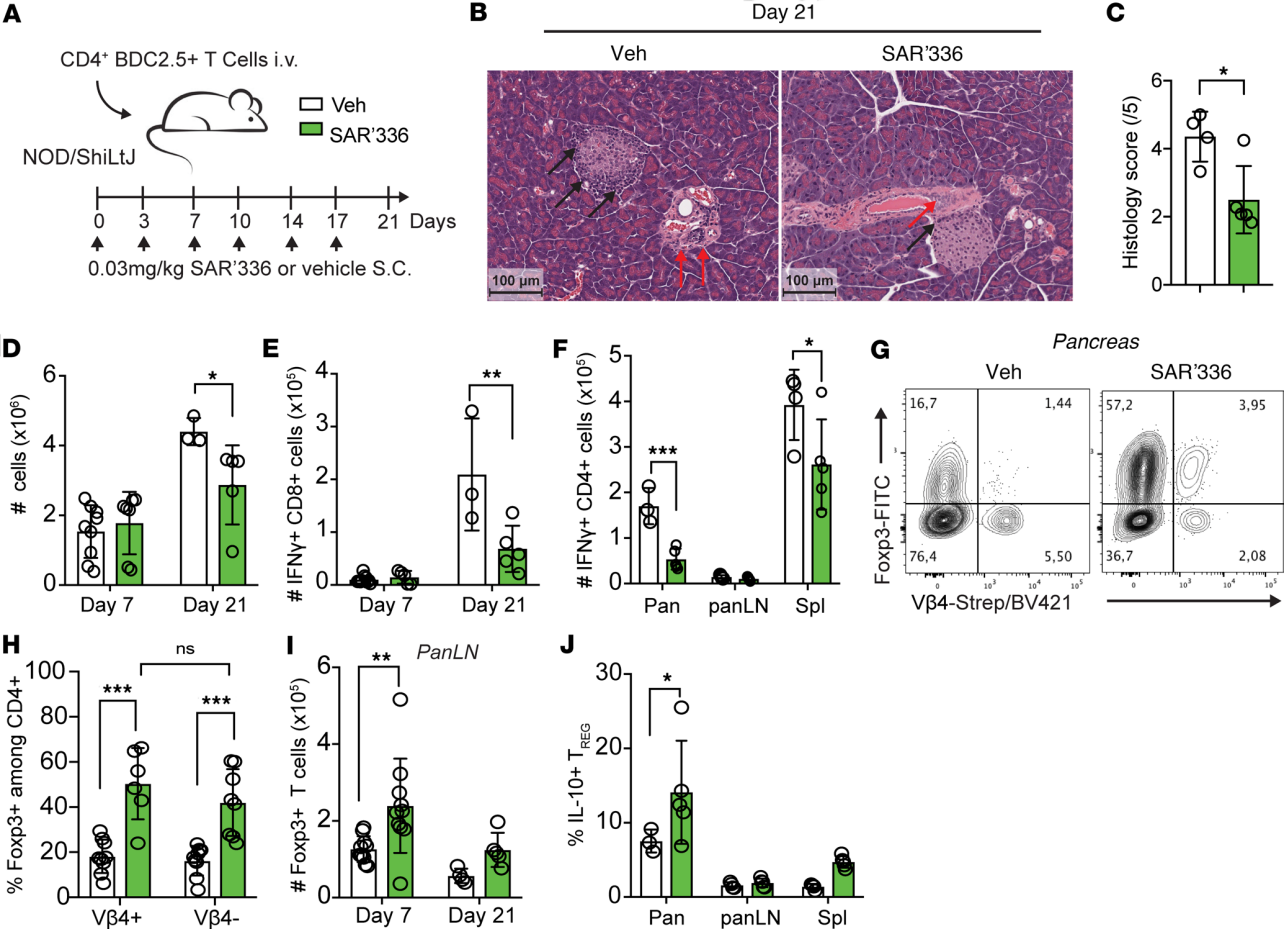
SAR’336 minimizes cytotoxic responses and expands IL-10–producing, antigen-specific Tregs in the pancreas. (**A**) CD4^+^ T cells were isolated from female NOD BDC2.5 mice, and 3 × 10^5^ cells were adoptively transferred i.v. into female NOD mice. A total of 0.03 mg/kg of SAR’336 was administered s.c. twice a week up to 21 days. Lymphocytes from the spleen, pancreatic LNs (panLN), and pancreas were collected at day 7 (*n* = 7–8/group) and day 21 (*n* = 3–5/group). Data compiled from 2 distinct experiments. (**B**) Representative histology slide of the pancreas at day 21 (hematoxylin and eosin). Black arrows show β-islet infiltration while red arrows point to perivascular infiltration of immune cells. (**C**) Histology score at day 21 posttransfer. Adapted from Papaccio et al. 2000 (86). 1 = infiltrates in small foci at the islet periphery; 2 = infiltrates surrounding the islets (peri-insulitis); 3 = intraislet infiltration < 50% of the islet, without islet derangement; 4 = extensive infiltration over 50% of the islet, cell destruction, and prominent cytoarchitectural derangement; 5 = complete islet atrophy and β cell loss. (**D**) Total number of cells isolated from the pancreas at day 7 and 21. (**E**) Number of IFN-γ–producing CD3^+^CD8β^+^ T cells (CD8^+^ T cells) in the pancreas. (**F**) Number of IFN-γ–producing CD3^+^CD4^+^ T cells in distinct organs at day 21 after transfer. (**G**) Representative flow cytometry of the expression of FoxP3 and Vβ4 in the pancreas at day 21 after transfer. (**H**) Frequency of FoxP3^+^CD4^+^ T cells among Vβ4^+^ and Vβ4^–^ T cells at day 21 after transfer. (**I**) Number of FoxP3^+^CD4^+^ T cells in the pancreatic lymph node at day 7 and 21 after transfer. (**J**) Frequency of IL-10–producing CD4^+^FoxP3^+^ Tregs in the pancreas (Pan), pancreatic LNs (panLN), and spleen (Spl) at day 21 after transfer. One way-ANOVA. Tukey’s correction. *P < 0.05; **P < 0.01; ***P < 0.001.

Since we observed that SAR’336 induces an expansion of Tregs in the pancreas of NOD mice, we next investigated whether it prevents the development of immune cell infiltration of β islets before the onset of diabetes (hyperglycemia). To this end, we used a model of adoptive transfer of antigen-specific BDC2.5 CD4^+^ T cells in prediabetic NOD mice to synchronize the onset of insulitis and better trace donor cells throughout diabetes development in NOD mice (45). CD4^+^ T (3 × 10^5^) cells were isolated from NOD BDC2.5 (Vβ4^+^) female mice and transferred i.v. into young adult female NOD mice (45) that received 0.03 mg/kg SAR’336 twice a week, an amount established previously as 1/10 of maximal dose (27) (Figure 3A). To better assess the early and late events occurring in the pancreas, pancreatic and systemic T cell populations were isolated at early (day 7) and late (day 21) stages of insulitis but before diabetes onset (Supplemental Figure 3). First, at a late time point, we observed less infiltration into the pancreatic islets in mice treated with SAR’336 relative to vehicle (Figure 3, B and C). Both the number of total cells and the number of IFN-γ–producing CD8^+^ T cells isolated from the pancreas at day 21 after transfer were lower in SAR’336-treated mice (Figure 3, D and E). Concomitantly, the number of IFN-γ^+^CD4^+^ T cells was reduced in the pancreas and the spleen of treated mice (Figure 3F). Administration of the engineered molecule induced an increase in the frequency of FoxP3^+^ Tregs among both recipient (Vβ4^–^) and donor (Vβ4^+^) CD4^+^ T cells in the pancreas (Figure 3, G and H) but not in the numbers of local FoxP3^+^ Tregs (Supplemental Figure 4A), resulting in a significantly lower ratio of IFN-γ–producing Th1 cells to Tregs in the pancreas at day 21 (Supplemental Figure 4B). Concomitantly, SAR’336 drove the accumulation of FoxP3^+^ Tregs in the pancreatic LN (Figure 3I), which have been shown to suppress inflammatory T cells prior to their migration to the pancreas (46, 47) by, notably, producing IL-10 (48). We observed that SAR’336 significantly increased the frequency of IL-10^+^ Tregs on day 21 (Figure 3J), highlighting the ability of SAR’336 to prevent the establishment of a cytotoxic immune response and promote the accumulation of IL-10^+^ Tregs in the pancreas.

### SAR’336 promotes the accumulation of antigen-specific IL-10^+^ Tregs in β islets.

**Figure 4:**
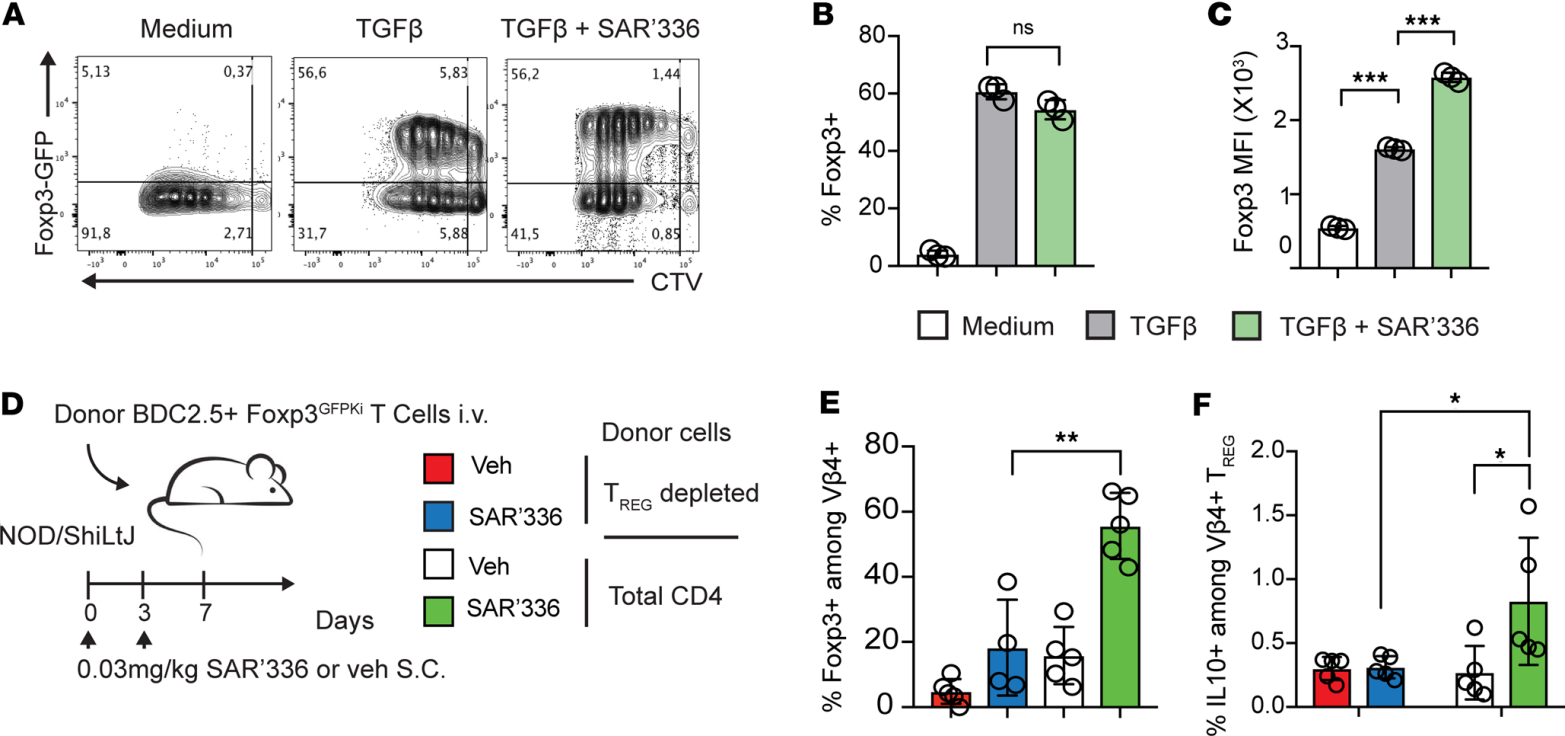
SAR’336 promotes the migration of antigen-specific, IL-10^+^ Tregs. (**A**–**C**) Splenic CD4^+^GFP^–^ Teffs were CTV-labeled and activated in the presence of APCs and soluble anti-CD3 (1 μg/mL) with 5 ng/mL murine TGF-β and 1 μg/mL of SAR’336 for 72 hours. Representative flow cytometry of CTV and FoxP3-GFP expression. Representative of 2 distinct experiments. (**B**) Frequency of FoxP3^+^ among total live CD4^+^ cells. (**C**) Geometric MFI of FoxP3 among total CD4^+^ T cells. Seventy-two hours. Two-way ANOVA. ***P < 0.001. (**D**–**F**) CD4^+^GFP^–^ (Treg depleted) or total CD4^+^ T cells were isolated from female BDC2.5 FoxP3^GFPki^ NOD mice, and 3 × 10^5^ cells were adoptively transferred i.v. into female NOD mice. At days 0 and 3 after transfer, 0.03 mg/kg of SAR’336 was administered s.c. One-way ANOVA. Tukey’s correction. *P < 0.05, **P < 0.01.

Since we observed that SAR’336 promoted Treg expansion, we next investigated whether the Tregs found in the pancreas were generated locally or migrated to the site upon inflammation. While the majority of FoxP3^+^ Tregs found in tissues originate from thymic selection (tTregs) and migrate to the tissue, a subset of local Tregs develop from the TGF-β–dependent induction of FoxP3 (49, 50) in naive T cells, so-called peripheral Tregs (pTregs) (51). IL-2 plays a role in the accumulation of pTregs but is not directly involved in the induction of FoxP3 (52, 53), suggesting a similar mechanism for SAR’336. To verify this, CD4^+^FoxP3^–^ cells isolated from B6.FoxP3^GFPki^ reporter mice were activated in the presence of recombinant TGF-β to generate FoxP3^+^ T cells. SAR’336 did not further contribute to the generation of FoxP3^+^ T cells in vitro (Figure 4, A and B). Nonetheless, once the cells gained FoxP3 expression, SAR’336 promoted the overall level of expression of the transcription factor (MFI) (Figure 4C). To investigate whether the protective effect of SAR’336 was dependent on migrating antigen-specific Tregs, we transferred donor BDC2.5^+^CD4^+^ T cells containing Tregs (“Total CD4”) or devoid of Tregs (“Treg depleted”) into NOD recipient mice (Figure 4D and Supplemental Figure 5A). While SAR’336 promoted the generation of pTregs from donor Vβ4^+^CD4^+^ T cells in the pancreatic LN (Supplemental Figure 5B), this was not observed in the pancreas (Figure 4E). Importantly, we did not observe IL-10 production from the donor Vβ4^+^ pTregs when compared with the group that received total BDC2.5^+^CD4^+^ T cells (Figure 4F), verifying that the protective Tregs expanded by SAR’336 originated from the transferred antigen-specific Tregs. Collectively, these results suggest that SAR’336 promotes the expansion of migrating Tregs rather than local pTregs to control inflammation in the pancreas.

### SAR’336 promotes GATA3 expression in expanding pancreatic Tregs.

**Figure 5:**
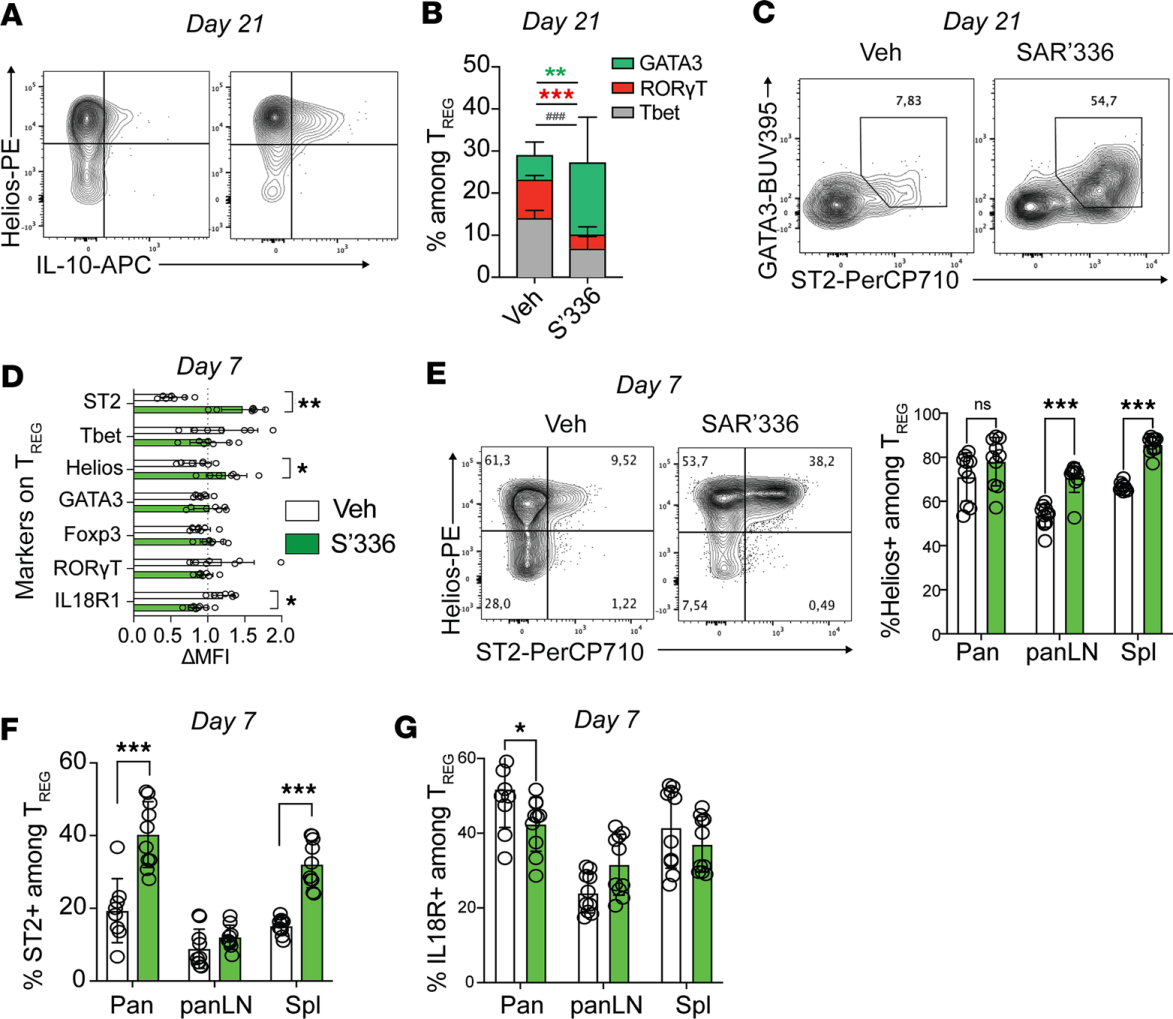
SAR’336 expands Helios^+^ST2^+^ Tregs in the pancreas. (**A**) Representative flow cytometry plot of IL-10 and Helios expression among CD4^+^FoxP3^+^ T cells in the pancreas of vehicle- (left) and SAR’336-treated (right) mice at day 21. (**B**) Intracellular expression of master transcription factors RORγT (red), T-bet (gray), and GATA3 (green) in CD4^+^FoxP3^+^ T cells isolated from the pancreas at day 21 after adoptive transfer. Individual Student’s *t* test between markers. **P < 0.01; ***P < 0.001; ^###^*P* < 0.001. (**C**) Representative flow cytometry plot of the expression of GATA3 and ST2 in CD3^+^CD4^+^FoxP3^+^ T cells isolated from the pancreas of vehicle- (veh) or SAR’336-treated mice at day 21. (**D**) Differential expression of ST2, GATA3, T-bet, RORγT, Helios, FoxP3, and IL-18R1 on CD4^+^FoxP3^+^ Tregs (gMFI/mean gMFI in both groups) from the pancreas at day 7. (*n* = 7–8/group.) Two-way ANOVA. *P < 0.05; **P < 0.01. (**E**) Representative flow cytometry of Helios and ST2 expression on pancreas-isolated CD4^+^FoxP3^+^ T cells at day 7 after transfer and frequency of Helios^+^ Tregs in the pancreas, panLN, and spleen at day 7 after transfer. Two-way ANOVA. ***P < 0.001. (**F**) Frequency of ST2^+^ among FoxP3^+^ Tregs in the pancreas, panLN, and spleen at day 7 after transfer. Two-way ANOVA. ***P < 0.001. (**G**) Frequency of IL-18R^+^ among FoxP3^+^ Tregs in the pancreas, panLN, and spleen at day 7 after transfer. Two-way ANOVA. *P < 0.05.

Although their transcriptional program is largely driven by the master transcription factor FoxP3 (19), Tregs acquire additional master transcription factors associated with Th cells, including GATA3, RORγT, and T-bet, to promote unique aspects of their migration, survival, and function during inflammation (20). IL-2 can directly orchestrate the transcriptional trajectory of Tregs (54, 55), notably, by promoting the generation of Tregs that express GATA3 (30) and Helios (55). Indeed, in the NOD T cell transfer experiment (Figure 3), we observed that IL-10^+^ Tregs SAR’336 generated (Figure 3J) in the pancreas also expressed the transcription factor Helios (Figure 5A). By day 21 after T cell transfer, SAR’336 promoted significantly more GATA3^+^ and less RORγT^+^ and T-bet^+^ Tregs in the pancreas relative to vehicle-treated mice (Figure 5B). Importantly, these pancreatic GATA3^+^ Tregs emerged from a population of ST2^+^ Tregs (Figure 5C), verifying a link between the expression of this transcription factor and the receptor for IL-33 (55, 56).

Since there is evidence that IL-33 drives accumulation of highly suppressive GATA3^+^ Tregs in the pancreas (33), we asked whether SAR’336 promoted the expansion of GATA3^+^ Tregs by directly inducing ST2 on Tregs. Using an in vitro approach, we observed that SAR’336 promoted the generation of ST2^+^ Tregs (Supplemental Figure 6A). Interestingly, SAR’336 also promoted the accumulation of GATA3^+^ Tregs in the presence of distinct T cell–polarizing conditions (Supplemental Figure 6, B and C), namely Th1 (IL-12 + IL-18), Th2 (IL-4 + IL-33), or Th17 (IL-6 + TGF-β + IL-1β) (54), suggesting that SAR’336 can influence the differentiation of these cells in various inflammatory environments. Indeed, SAR’336 suppressed T-bet expression in Tregs exposed to IL-12 (Supplemental Figure 6, D and E), but not in Teffs (Supplemental Figure 6F), and inhibited RORγT expression in Tregs exposed to IL-6, TGF-β, and IL-1β (Supplemental Figure 6, G and H), verifying a selective effect of SAR’336 on Tregs. Collectively, these observations reveal that, in addition to promoting GATA3 expression, SAR’336 prevents the generation of T-bet^+^ and RORγT^+^ Tregs.

Finally, we asked whether SAR’336 influenced the tissue differentiation of early infiltrating Tregs or whether this process happened in the late stages of inflammation. At day 7 after the initial dose, we observed that SAR’336 increased the expression (MFI) of ST2 and Helios and reduced the expression of the Th1-associated IL-18R1, the receptor for IL-18, in pancreatic FoxP3^+^ Tregs (Figure 5D). We did not observe an increase in GATA3 expression in Tregs by day 7 (Figure 5D), suggesting that SAR’336 initially promotes the expression of ST2, which, in turn, favors the accumulation of GATA3^+^ Tregs by day 21. While the frequency of Helios^+^ Tregs was not increased in the pancreas (Figure 5E), we observed a significant increase in the frequency of ST2^+^Helios^+^ Tregs in the pancreas and spleen by day 7 after administration of SAR’336 (Figure 5F) and a reduction in the frequency of IL-18R^+^ Tregs (Figure 5G), highlighting the capacity of SAR’336 to promote the specific generation of protective (33) ST2^+^ Tregs in the early phase of insulitis in NOD mice.

## Discussion

Polymorphisms in genes that reduce the sensitivity of FoxP3^+^ Tregs to IL-2 are particularly overrepresented in patients with T1D, leading to aberrant Treg function and exacerbated autoimmune responses (57–59). This makes it challenging to design a dosing scheme for aldesleukin to target patients’ Tregs, as increasing aldesleukin dose levels or repeat dosing augments the risk of promoting rather than suppressing ongoing pathogenic autoimmune responses in T1D-susceptible individuals (60). A clinical trial demonstrated that it is particularly difficult to design an aldesleukin dose regimen in patients with T1D to obtain a sustained expansion of circulating CD4^+^CD25^+^CD127^lo^ Tregs without fueling the expansion of disease-promoting Teffs, NK cells, and eosinophils (61). In essence, rhIL-2 lacks an appropriate therapeutic index for treatment of autoimmune conditions, and new approaches using muteins of IL-2 are being investigated. Here, our results demonstrate how a CD25-biased IL-2 mutein, SAR’336, selectively signals Tregs in vitro and in vivo.

A major hurdle to the use of IL-2 and its muteins is that, while Tregs readily express high levels of CD25 at the steady state, activated Teffs also upregulate CD25 following cell activation (62). Using a model to track the expansion of antigen-specific T cells in vivo (63, 64), we show that pegylated SAR’336 can selectively target highly sensitive CD25^hi^ Tregs over antigen-specific (Tet^+^) Teffs when administered at relatively low dose levels (<0.3 mg/kg). Interestingly, we observed a 20% protection over control mice (vehicle alone), a disease incidence at day 21 that is well documented in this model (63, 64). At the chosen early (day 7) and late (day 21) time points before diabetes (63, 64), SAR’336 preferentially promoted Treg expansion while circumventing effects on recently activated CD25^+^ Teffs. This effect may be due to the pegylation of SAR’336 at the H16 position (27), since an H16 mutation disrupts the signal provided to CD25^+^ Teffs more than it does on CD25^+^ Tregs (9). Here, although we did observe a dose-dependent effect of SAR’336 in vivo and in vitro, our approach did not allow for the establishment of a therapeutic index, and further dose ranging studies are required. Nonetheless, we were able to capture how the unique design of SAR’336 allows it to efficiently target CD25^+^ Tregs even in the presence of CD25^+^ Teffs.

In addition to its specificity in expanding Tregs in lymphoid tissues, we demonstrate that systemic administration of SAR’336 can act in a nonlymphoid tissue to prevent inflammation. A single dose of SAR’336 preferentially expanded Tregs over NK cells, cytotoxic T cells, and Teffs in the pancreas of NOD mice. Moreover, we show that SAR’336 prevents autoimmune cell infiltration in the pancreas following transfer of islet-specific BDC2.5^+^ T cells to NOD mice (40–42). This is consistent with reports that delivery of IL-2 or IL-2 muteins to the pancreas can prevent spontaneous or induced diabetes in NOD mice (2, 14) and in a model of BDC2.5 T cell transfer (65). SAR’336 lowered the numbers of infiltrating Th1 and CD8^+^ T cells, expanded IL-10^+^ Tregs in the pancreas of mice, and reduced β islet destruction. In addition to these tissue-specific effects, SAR’336 promoted the expansion of Tregs in pancreatic LNs. Tregs found in draining LNs have been found to be highly suppressive (66) and likely contribute to the dampening of insulitis in our model. The clinical relevance of these observations remains to be assessed, as we stopped the experiment before the full onset of glycemia, and further investigation into the outcome of disease is warranted. Nonetheless, these results provide evidence that SAR’336 can modify the pancreatic and lymphatic immune landscape to favor antigen-specific, suppressive Treg responses to prevent T cell–mediated β islet damage.

Since Tregs of distinct developmental origin can be found in tissues, we next attempted to determine the origin of these cells in our model. Our results show that the effect conferred by SAR’336 depended on the presence of migrating BDC2.5^+^ Tregs, rather than on the induction of FoxP3 expression in BDC2.5^+^ Teffs, providing evidence that tTregs, not pTregs, were expanded by SAR’336. Both tTregs and pTregs are highly suppressive cells that share very similar phenotypes despite different developmental pathways (67). Despite this, they do not share the same TCR repertoire (67), playing nonredundant roles in the prevention of T1D (68, 69). Indeed, pTregs can delay disease progression in the NOD model, though, contrary to tTregs (69), their role in the control of insulitis is limited (68). In our experiments, we did observe that SAR’336 can promote the in vitro and in vivo expansion of pTregs, which can also contribute to local suppression through distinct mechanisms. Another potential piece of evidence that tTregs are preferentially expanded by SAR’336 is the observation that it promoted the expansion of Tregs expressing the transcription factor Helios, a marker of highly suppressive and stable Tregs (70) that is particularly, but not exclusively, expressed by tTregs (51, 71). The preferential expansion of Helios^+^ over Helios^–^ Tregs may lie in Helios’s promotion of IL-2/STAT5 signaling and CD25 expression (38), making Tregs more sensitive to relatively low doses of IL-2. This is in line with a report that demonstrated that low-dose aldesleukin preferentially expanded Helios^+^ Tregs in patients with T1D (72), providing a rationale for the use of low-dose aldesleukin to reinvigorate exhausted Helios^lo^ Tregs in patients with T1D (73). Thus, while the immediate protective effect of SAR’336 in this model may be conferred by promoting the expansion and function of antigen-specific tTregs in the pancreas, the role of locally induced pTregs remains to be determined.

Finally, while characterizing the phenotype of Tregs induced by SAR’336, we observed an increase in cells expressing the IL-33 receptor, ST2. In recent years, IL-33 was shown to potentiate the proliferation and function of tissue-derived Tregs (55, 56) and is currently considered a potential therapy to increase Treg activity in autoimmune diseases (74, 75). In particular, IL-33 improves the in vitro suppressive capacity of Tregs isolated from patients with T1D (76). Murine models reveal that IL-33 drives the expansion of highly suppressive GATA3^+^CTLA4^+^ Tregs that, in turn, prevent the onset of diabetes (33). This is in line with reports showing that IL-33 promotes the expansion of Tregs that express GATA3 (37, 56, 77), a transcription factor that supports the FoxP3 transcriptional program (78). On the other hand, SAR’336 led to a decrease in the abundance of T-bet^+^ and RORγT^+^ Tregs in the pancreas. Expression of T-bet helps slow the progression of diabetes in NOD mice (32), as it is required for Tregs to express the chemokine CXCR3 that enables migration to the pancreas (79). However, the fate and function of T-bet^+^ Tregs in the pancreas remains ill-defined, and it is possible that SAR’336 orchestrates the expression of GATA3 in T-bet^+^ Tregs (80) by favoring IL-33 over IL-18 signals (54). Similarly, RORγT^+^ Tregs with suppressive capacity have been identified in the pancreas of NOD mice (81), albeit with the caveat that they are likely to lose FoxP3 expression and contribute to inflammation in the presence of IL-6 (81). Moreover, we observed that SAR’336 promoted the accumulation of IL-10–secreting Tregs in the pancreas rather than in their counterparts from secondary lymphoid organs. This suggests that Tregs in the pancreas have acquired a tissue-adapted ST2^+^IL-10^+^ effector phenotype when compared with Tregs in circulation or in secondary lymphoid organs. Indeed, there is extensive literature detailing the many epigenetic and transcriptional changes pancreatic Tregs undergo in the NOD mouse, including overexpression of *Il10* (82). Here, we hypothesize that SAR’336, through the phosphorylation of STAT5, promotes IL-10 production in pancreatic Tregs (83). It remains to be understood, however, if SAR’336 acts by facilitating the tissue differentiation of pancreatic Tregs — prior to their egress into the pancreas — or if it targets ST2^+^ Tregs in the pancreas, acting in concert with local IL-33. Nonetheless, these observations on the tissue adaptation of Tregs provide new insights on the mechanisms by which SAR’336 supports the establishment of a stable population of functional Tregs.

Collectively, we show how SAR’336, a CD25-biased SYNTHORIN IL-2 mutein, preferentially promotes the expansion and influx of highly adapted Tregs to pancreatic islets. In doing so, SAR’336 promotes the induction, expansion, and accumulation of IL-10–secreting FoxP3^+^ Tregs, with the potential to reduce the severity of insulitis and downstream T1D induction in NOD mice. Although further investigation is indicated to better understand the impact of this technique on the control of diabetes, this work provides mechanistic insights into the antiinflammatory and therapeutic effects of biased, engineered IL-2 molecules and a conceptual basis for their use as therapeutic agents in select autoimmune and inflammatory diseases.

## Methods

### Sex as a biological variable.

For the NOD.TCRα and NOD BDC2.5 experiments, female mice were used as they display earlier pancreatic islet inflammation compared with males (84). For all the in vitro experiments, primary immune cells from female or male mice were used, as sexes were not considered as a biological variable.

### Mice.

B6.FoxP3^GFPki^ mice were provided by Alexander Rudensky (85) and bred in-house. Female 8- to 12-week-old NOD/ShiLtJ (strain 001976), NOD.TCRα [NOD.129P2(C)-Tcratm 1Mjo/DoiJ; strain 004444], and NOD BDC2.5 (NOD.Cg-Tg TcraBDC2.5, TcrbBDC2.5 1Doi/DoiJ; strain 004460) mice were obtained from Jackson Laboratories. Donor NOD BDC2.5 FoxP3^GFPki^ were obtained from crossing B6.FoxP3^GFPki^ into NOD BDC2.5 for more than 12 generations (48).

### Purification of T cell subsets.

After isoflurane/CO_2_ euthanasia, the spleens of donor NOD BDC2.5 mice were isolated and kept in complete RPMI 1640 + 10% FBS (Wisent) on ice. The remaining blood cells were lysed using an in-house made ammonium-chloride-potassium buffer, as previously described (55). Cells from pancreatic LNs were isolated mechanically as for spleens. To collect the cells from the pancreas, the organs underwent an initial enzymatic digestion in collagenase IV (1 mg/mL; MilliporeSigma) and DNase I (0.005 mM; MilliporeSigma) for 45 minutes followed by a round of dissociation with a nonenzymatic cell dissociation buffer (Gibco, Thermo Fisher Scientific) for 5 minutes at 37°C. The tube contents were then filtered in a 70 μm mesh and the cells counted using trypan blue (Gibco, Thermo Fisher Scientific). For in vitro culture, splenic cells were incubated for 20 minutes with anti-mouse CD4 magnetic beads (Miltenyi Biotec). CD4^+^ T cells were enriched through an autoMACS (Miltenyi Biotec) and stained with a noncompeting clone of anti-mouse CD4 (RM4.5; Thermo Fisher Scientific) before sorting through a FACSAria (BD Biosciences). For in vitro experiments, CD4^+^ cells were further sorted into GFP^+^ and GFP^–^ T cells (B6.FoxP3^GFPki^ NOD BDC2.5 FoxP3^GFPki^) or CD25^lo^ and CD25^hi^ (NOD BDC2.5) using FACSAria (BD Biosciences) at more than 99% purity.

### Cell culture.

To investigate the in vitro effects of SAR’336 (Sanofi) on Tregs, purified CD4^+^GFP^+^ Tregs (GFP^+^, 50 × 10^3^) from B6.FoxP3^GFPki^ female mice were activated in 96-well, flat-bottomed (0.2 mL) plates previously coated with α-CD3 (3 μg/mL) and α-CD28 (2 μg/mL), in the presence of rhIL-2 (1 μg/mL; Chiron Corporation) or SAR’336 (1 μg/mL) in RPMI supplemented with 10% FBS at 37°C for 72 hours. The Treg induction assay was performed by sorting CD4^+^GFP^–^ (Teffs) from B6.FoxP3^GFPki^ mice. The cells were then labeled with CellTrace Violet (Thermo Fisher Scientific) and activated in 96-well, flat-bottomed (0.2 mL) plates previously coated with α-CD3 (3 μg/mL) and α-CD28 (2 μg/mL) in the presence or absence of rhIL-2 (1 μg/mL) or SAR’336 (1 μg/mL) and TGF-β (5 ng/mL; Novoprotein Scientific). For the polarization assays, 25 × 10^3^ purified CD4^+^GFP^–^ cells (Tregs) and 50 × 10^3^ CD4^+^GFP^–^ cells (Teffs) from B6.FoxP3^GFPki^ mice were plated in 96-well, flat-bottomed plates together with 2.0 × 10^5^ irradiated feeder cells (CD4^–^ fraction of the MACS) and soluble αCD3 (1 μg/mL) in the presence of IL-12 (10 ng/mL), IL-4 (10 ng/mL), or a combination of IL-6 (50 ng/mL) and TGF-β (1 ng/mL; Novoprotein Scientific). Freshly sorted CD4^+^ T cells were labeled with CellTrace Violet. The cells were then cocultured at different ratios in the presence of mitomycin C–treated CD4^–^ cells (antigen-presenting cells) and 0.03 μg/mL α-CD3 (clone OKT3; Thermo Fisher Scientific) in the presence or absence of SAR’336 (Sanofi).

### In vivo injections of IL-2 mutein.

Female 8- to 10-week-old NOD/ShiLtJ or NOD BDC2.5 mice received 0.3 mg/kg of SAR’336 s.c. and were sacrificed at days 2 and 4 after injection. The mice were euthanized by isoflurane/CO_2_, and the PBMCs were collected through intracardiac puncture in K2 EDTA tubes. A portion of the pancreas was isolated and fixed in 10% formalin (MilliporeSigma) for paraffin embedding, followed by hematoxylin and eosin stain. The pancreas histology score at day 21 after transfer was adapted from Papaccio et al. (86): 1 = infiltrates in small foci at the islet periphery; 2 = infiltrates surrounding the islets (peri-insulitis); 3 = intraislet infiltration < 50% of the islet, without islet derangement; 4 = extensive infiltration over 50% of the islet, cell destruction, and prominent cytoarchitectural derangement; 5 = complete islet atrophy and β cell loss. The spleens, inguinal LNs, and remaining pancreas were collected. The pancreas was cut into 5 mm pieces and digested in RPMI 1640 with 5% FBS containing collagenase D (0.5 mg/mL) in the presence of DNase I (0.005 μM) for 30 minutes at 37°C and then processed mechanically through a 70 μm cell strainer. The LNs and the spleen were mechanically processed using the back of a syringe plunger through a 70 μm cell strainer.

### T cell transfer NOD model of T1D.

For total CD4^+^ T cell transfer, CD4^+^ T cells from NOD BDC2.5 donor female mice were enriched and sorted as described above. For the Treg-depleted experiment, purified total CD4^+^ T cells or CD4^+^GFP^–^ cells from NOD BDC2.5 FoxP3^GFPki^ mice were sorted on the day of the transfer, then kept in PBS on ice until tail vein injection. For the 4-day study, 5 × 10^6^ CD4^+^ (NOD BDC2.5) cells were injected (200 μL/mouse) into 8- to 10-week-old female NOD/ShiLtJ that received a single dose of 0.01, 0.1, or 0.3 mg/kg of SAR’336. In the long-term diabetes study 3 × 10^5^ CD4^+^ T cells or CD4^+^GFP^–^ (Treg depleted) were adoptively transferred into the tail vein of 8- to 10-week-old female NOD/ShiLtJ mice. The mice received 0.03 mg/kg of SAR’336 every 3–4 days starting at day 0. The spleen, pancreatic LNs, and pancreas were collected and processed as described above.

### Cytokine assay.

To assess cytokine production, the isolated cells were exposed to PMA (MilliporeSigma), ionomycin (MilliporeSigma), monensin (GolgiStop; BD Biosciences), and Brefeldin A (Thermo Fisher Scientific) at manufacturer-recommended concentrations for 3 hours.

### Flow cytometry.

Single-cell suspensions were stained with the following fluorescence-conjugated mAbs: α-CD3 BUV737 (17A2; BD Biosciences), α-CD4 Alexa Fluor 700 (RM4.5; Thermo Fisher Scientific), α-CD8b BV510 (H35-17.2) (BD Biosciences), α-ST2 PerCP710 (RMST2-2; Thermo Fisher Scientific), α-IL18R1 APC or PECy7 (P3TUNYA; Thermo Fisher Scientific), α-KLRG1 BUV395 (2F1, BD Biosciences), α-CD25–PECy7 (PC61; BD Biosciences), α-FoxP3–FITC (FJK-16s; Thermo Fisher Scientific), α-IL17A–APC (eBio17B7; Thermo Fisher Scientific), α–IFN-γ–BUV737 (XMG1.2; BD Biosciences), α–IL-10 APC (JES5-16E3; Thermo Fisher Scientific), α-NKp46-BV650 (29A1.4; BioLegend), α-CD19-APC-Cy7 (ID3; BD Biosciences), α-RORγT BV786 (Q31-378, BD Biosciences), α-GATA3 Alexa Fluor 647 or BUV395 (BD Biosciences), α-Helios Pacific Blue or PE (22F6; BioLegend), α-TCR Vβ4-biotin (KT4; BD Biosciences), Streptavidin conjugate PE-Cy7, and CD45.2 APC–eFluor 780 (104, Thermo Fisher Scientific). The tetramer BDC2.5 mimetope (TS-M737) was obtained from MLB International Corporation. Nonviable cells were excluded using fixable viability dye eFluor 780 or 506 reagents (Thermo Fisher Scientific). Data were acquired using a FACSFortessa X-20 flow cytometer (BD Biosciences) or Attune NxT flow cytometer (Thermo Fisher Scientific) and analyzed using FlowJo version 9 software (BD Biosciences).

### Statistics.

For all experiments, the mean and standard deviation are shown. Multiple comparisons were tested using a 2-way ANOVA with Holm-Šídák correction. One-way ANOVA using the Tukey post hoc test was used for comparison of all individual means within a figure. For single comparisons unpaired 2-tailed Student’s *t* test was used with the *P* value expressed in the figure legend. All statistical analysis was performed with GraphPad Prism version 9 software. *P* < 0.05 was considered statistically significant.

### Study approval.

Animal housing and welfare comply with the US Department of Agriculture’s Animal Welfare Act (9 CFR Parts 1, 2, and 3) and the Canadian Council on Animal Care where applicable. The study was approved by the animal compliance committee of McGill University. All procedures performed involving animals were conducted following an approved IACUC protocol, in accordance with the ethical standards of McGill University or practice at which the studies were conducted.

### Data availability.

The datasets generated or analyzed during the studies reported herein are available in the Supporting Data Values file provided in a single Excel sheet, and repeats and supporting data sets can be obtained from the corresponding author on reasonable request. The datasets generated during or analyzed during the studies reported herein do not make use of custom code or mathematical algorithms central to the conclusions.

## Author contributions

FA, CAP, and MFM established the study framework. FA and CAP wrote the paper. FA, NVA, GMLM, and MFM designed experiments. FA, GMLM, NS, and NVA performed the experiments. FA, GMLM, NS, and NVA analyzed the data. JMM and MP provided valuable input throughout the study. MEM helped design the initial studies that led to this work. All authors provided valuable input to the writing of the manuscript.

## Supplementary Material

Supplemental data

Supporting data values

## Version 1 12/20/2024

Electronic publication
