# Supplementary material for: The IL-2 SYNTHORIN molecule promotes functionally adapted Tregs in a preclinical model of type 1 diabetes
Source: JCI Insight. 2024 Dec 20;9(24):e182064. doi: 10.1172/jci.insight.182064 (PMC11665582; doi:10.1172/jci.insight.182064)
Supplement: Supplemental data [file jciinsight-9-182064-s102.pdf]

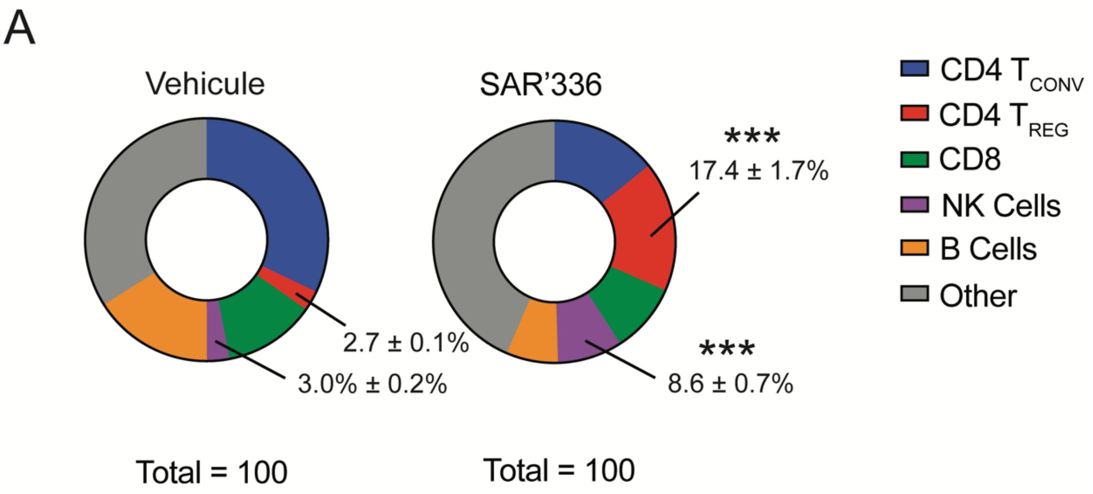

Supplemental Figure 1. SAR'336 expands circulating T_{REG} upon administration in vivo. (A) Representative piechart of the frequency of CD3+, conventional T cells (T_{CONV}), T_{REG}, CD8α+, NK and B cells in PBMCs in NOD mice between vehicule and SAR'336 treated at day 4 post-injection with 0.3mg/kg of SAR'336. Mean of expression shown (n=5/group). Two-way ANOVA. ***p<0.001

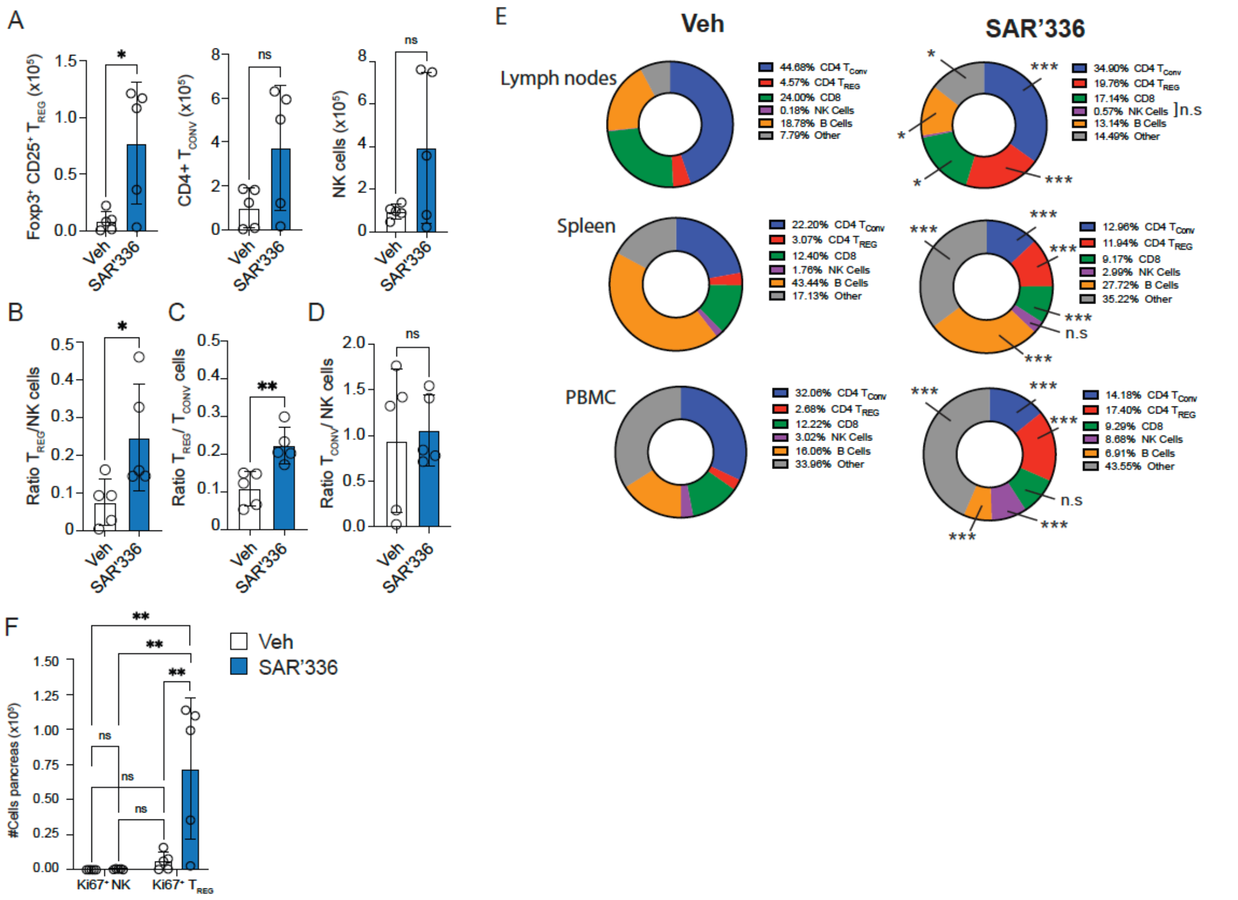

Supplemental Figure 2. SAR'336 preferentially expands circulating T_{REG} over NK and CD4+ T cells *in vivo*. NOD female mice were administered 0.3mg/kg of SAR'336 or the vehicule (veh) s.c. and cells from the blood (PBMCs), the spleen, inguinal and axillary LN (pLN) were collected at day 4 post-injection. (n=4-5/group) (A) Number of ($CD4^+$ $Foxp3^+CD25^+$) T_{REG} cells, $Foxp3^{NEG}$ $CD4^+$ T_{CONV} cells and ($NK1.1^+$ $CD3^-$) NK cells at day 4 in the spleen. (B) Ratio of T_{REG} over NK cells in the spleen, (C) Ratio of T_{REG} over T_{CONV} cells and (D) Ratio of T_{CONV} over NK cells at day 4. (E) Representative piechart of the frequency of CD3+, conventional T cells (T_{CONV}), T_{REG}, CD8α+, NK and B cells from total CD45+ cells isolated from lymph nodes, spleen and the blood (PBMC) in NOD mice between vehicule and SAR'336 treated at day 4 post-injection with 0.3mg/kg of SAR'336. Mean of expression shown (n=5/group). Two-way ANOVA. ***p<0.001. (F) Numbers (Count) of $Ki67^+$ ($NK1.1^+$ $CD3^-$) NK cells and $Ki67^+$ ($CD4^+$ $Foxp3^+CD25^+$) T_{REG} cells in the pancreas at day 4. One-way ANOVA, **p<0.01.

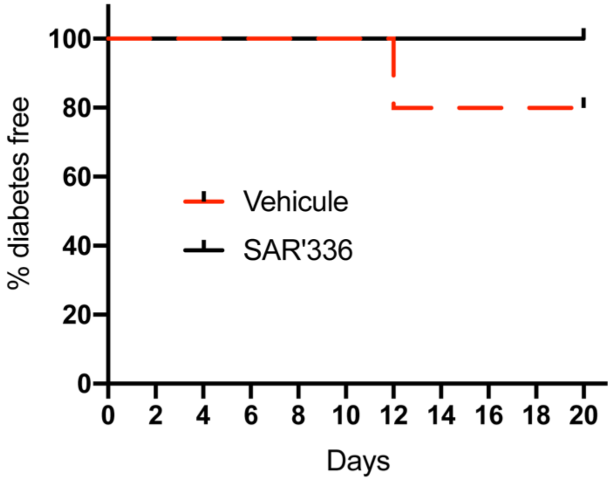

Supplemental Figure 3. Diabetes onset in NOD mice after 21 days.
3 x 10^5 $CD4^+$ T cells isolated from female ($V\beta4^+$) NOD.BDC2.5$^+$Foxp3^{GFPki} mice were adoptively transferred (i.v.) into 8–10-week-old female NOD/ShiLtJ mice to accelerate the onset of pancreatic infiltration into the pancreas. % Diabetes-free represented as a survival curve per group. % Diabetes-free mice established from daily blood glucose measurements. Diabetes threshold established as sustained high glucose >10 mmol/L; lowest value obtained 21mmol/L.

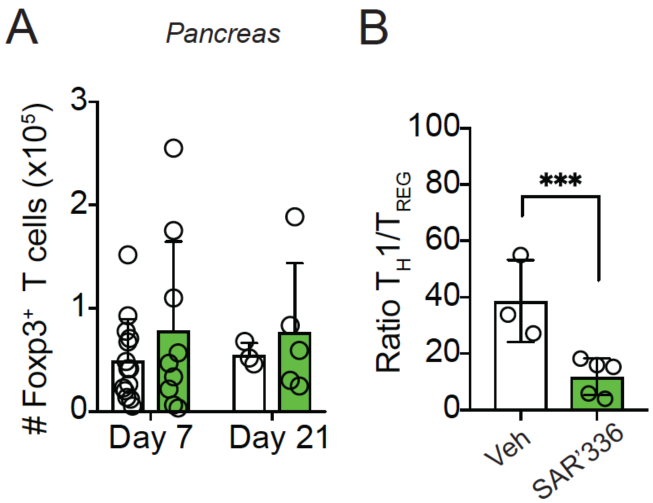

Supplemental Figure 4. T_{REG} cell numbers remain the same in the pancreas throughout treatment

$CD4^+$ T cells were isolated from female BDC2.5 NOD mice and $3x10^5$ cells were adoptively transfer i.v. into female NOD mice. 0.03mg/kg of SAR'336 was administered s.c. twice a week up to 21 days. Lymphocytes from the pancreas were collected at day 7 (n=7-8/group) and day 21 (n=3-5/group). Data compiled from two distinct experiments.

(A) T_{REG} cell numbers established by flow cytometry as $CD3^+$ $CD4^+$ $Foxp3^+$ cells.

(B) Ratio of the number of $IFN\gamma^+$ $CD4^+$ T cells (T_H1) over the number of $Foxp3^+$ $CD4^+$ T_{REG} in the pancreas at day 21.

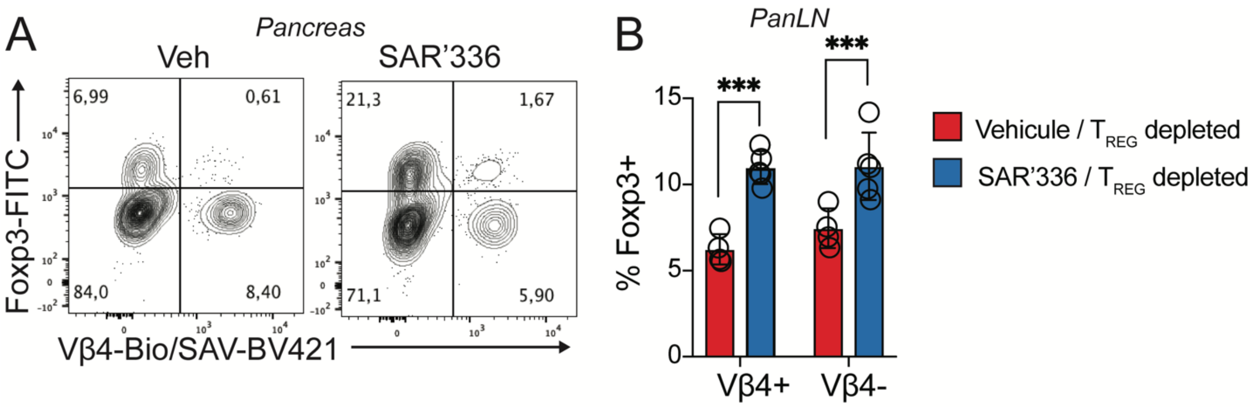

Supplemental Figure 5. SAR'336 promotes pT$_{REG}$ cell accumulation.
(A) Representative flow cytometry plot of Foxp3 and VB4 expression among $CD3^{+}CD4^{+}$ T cells in the pancreas at day 7.
(B) Frequency of $Foxp3^{+}$ among $VB4^{+}$ and $VB4^{-}$ $CD3^{+}CD4^{+}$ T cells in pancreatic LN. One-way ANOVA. Tukey post-hoc correction. **p<0.01; ***p<0.001.

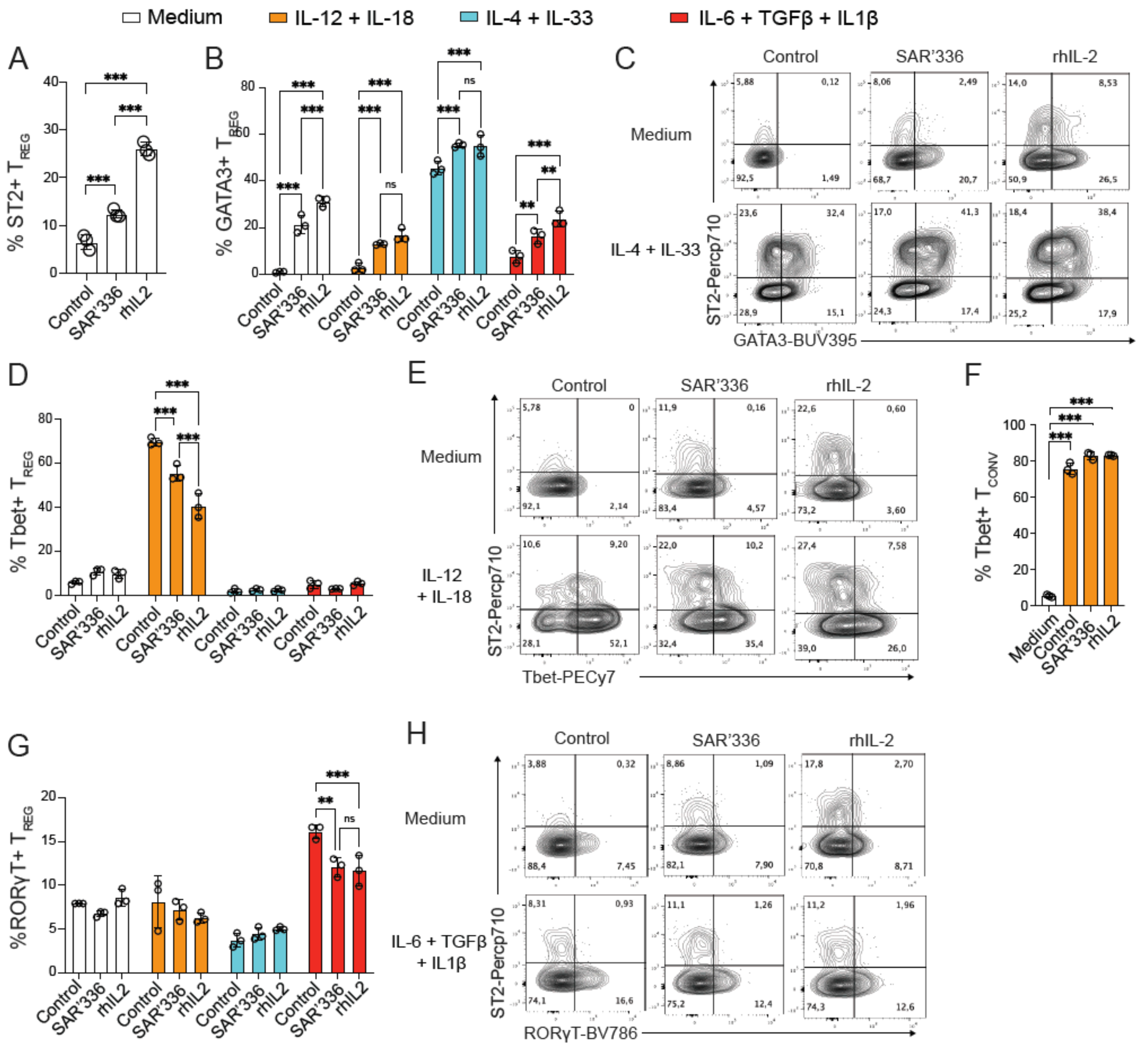

Supplemental Figure 6. SAR'336 promotes the induction of GATA3 in an IL-4 and IL-33 environment over Tbet and RORγT in activated T_{REG} cells *in vitro*.

Splenic $CD4^{+}Foxp3\text{-}GFP^{neg}$ CTV-labelled T_{EFF} cells and $Foxp3\text{-}GFP^{+}$ T_{REG} cells were co-cultured at a ratio of 1 T_{REG} : 4 T_{EFF} in the presence of APCs and soluble anti-CD3 (1 µg/ml) in medium (white bar), TH1 conditions IL-12 (10ng/ml) + IL-18 (10ng/ml) (orange bar), TH2 conditions IL-4 (10ng/ml) + IL-33 (10ng/ml) (Blue bar) or TH17 conditions IL-6 (10ng/ml) + TGFβ (1ng/ml) + IL-1β (50ng/ml) (red bar) +/- 1 µg/ml of SAR'336 for 72 hours. Two-way ANOVA. **$p<0.01$; ***$p<0.001$.

(A) Frequency of $ST2^{+}$ T_{REG} in medium with/without SAR'336 or rhIL-2 at 1µg/ml (B) Frequency of $GATA3^{+}$ T_{REG} in all conditions at 72 hours,

(C) Representative flow cytometry of the expression of ST2 and GATA3 in $CD4^{+}GFP^{+}$ CTV-negative T_{REG} cells at 72 hours.

(D) Frequency of $Tbet^+$ T_{REG} in all conditions at 72 hours (E) Representative flow cytometry of the expression of ST2 and Tbet in $CD4^+GFP^+$ CTV-negative T_{REG} cells at 72 hours.
(F) Frequency of $Tbet^+$ among T_{CONV}
(G) Frequency of $RORγT^+$ T_{REG} cells at 72 hours. (H) Representative flow cytometry of the expression of ST2 and RORγT in $CD4^+GFP^+$ CTV-negative T_{REG} cells at 72 hours. Two-way ANOVA. **$p<0.01$; ***$p<0.001$. Representative of 4 independent experiments.
